# Supplementary material for: 3 ′-5 ′ crosstalk contributes to transcriptional bursting
Source: Genome Biol. 2021 Feb 4;22:56. doi: 10.1186/s13059-020-02227-5 (PMC7860045; doi:10.1186/s13059-020-02227-5)
Supplement: Supplementary file 2 — Additional file 2 Review history. [file 13059_2020_2227_MOESM2_ESM.docx]

Review History

**First round of review**

**Reviewer 1**

**Are you able to assess all statistics in the manuscript, including the appropriateness of statistical tests used?**

Yes, and I have assessed the statistics in my report.

**Comments to author:**

The manuscript of Cavallaro et al. studies the role of 3' - 5' crosstalk, gene looping and reinitiation of pol II in transcriptional bursting. This is an interesting concept, as the molecular mechanisms of bursting are mostly unknown. The hypothesis that the authors try to address experimentally has been suggested before, but has so far not been experimentally shown. The authors use a combination of approaches, including transgene analysis upon perturbation, genome-wide analysis of existing datasets and modeling, to show that reinitiation of Pol II upon termination contributes to bursting. The data quality is high, with many replicates and carefully performed controls. However, I have strong concerns on the analysis, interpretation and conclusions drawn from this data.

The authors use several transgene reporters and cell lines to study the effect of polyA mutations. Similar cell lines have been used before to show that polyA mutation reduce transcription initiations, which the authors also reproduce. The evidence that transcription initiation is really affected is not performed by the present study, but mostly relies on a previous study (ref 32). The authors then perform flowFISH to determine the mean and noise of the construct, which they then fit to several (bursting) models to acquire the bursting parameters. In general, it has been inherently difficult to capture dynamic parameters from indirect static measurements such as smFISH data, which is also true for flow-FISH. My main concern is that polyA mutation, in addition to inhibiting transcription initiation, can significantly affect the stability of the transcript. If the readthrough transcripts are degraded much faster, or are not exported to the cytoplasm, this may significantly affect the distributions. I could not find any hints in the manuscript that the authors have addressed this option, or how it would affect their modelling results. I therefore doubt that the effect on transcriptional noise that is observed can fully be explained by changes in bursting parameters. The authors would need to carefully exclude the option that there are effects on degradation or export of mRNA from the nucleus in these mutants.

Apart from degradation, extrinsic noise can also affect the distribution, one of which is cell cycle. The authors indeed nicely show that the contribution of cell cycle on the noise is very small. It is however a bit surprising that their overall extrinsic noise is such a small fraction of the total transcriptional noise, as it has been found to be much higher in other systems. I wonder whether other extrinsic noise components, such as cell size, could be missed in the current setup.

Given these possible confounding effects of estimating transcription kinetics from such indirect measurements, I would find the results much more convincing if they were shown using more direct approaches. There are live-cell imaging assays available that could directly measure burst size and burst frequency in living cells, using MS2/PP7 transcription imaging, which would be compatible with the transgene approach.

In addition to my concerns about the flowFISH interpretation, both the present manuscript, and the cited article 32, do not show that loss of the polyA site reduces contact between the 5' and 3' of a gene (minor note: ref 32 is wrongly cited in the introduction as if it shows this). Although there may be crosstalk, it appears that the conclusion that gene loops are involved in this is still not sufficiently shown experimentally.

The authors continue to show that genes with more noise in scRNAseq data have more ChIA-PET interactions between 3' and 5' than genes with less noise. Although the effects are modest, but this is a conceptually interesting finding. I do wonder how specific this effect is for 5' - 3' interactions (loops), or if you would find similar higher interactions between the 5' and gene body. Given the recent insights from microC data (Hsieh et al, Mol Cell 2020), could it be possible that the promoter or (3' end) interacts along the entire gene, including the 3' end (as seen by the stripes in microC). From the current analysis, where the interaction of 5' and 3' are compared to random genomic locations with the same distance, this would not be clear. Would the authors see the same if the compared the interactions of promoter to gene body for high/low noise genes? If so, that would change the interpretation of their results.

The modeling approach is a nice addition, and shows that recycling of polymerases could contribute to bursting. Given my concerns with the interpretation of the experimental data, on its own this model does not prove that recycling occurs. Also, no other models without recycling are considered, and I wonder if there would be other models that would equally well explain the observed data.

Minor comments:

The hypothesis that liquid condensates are involved is interesting, but this is very much a developing field, where the role of phase separation in transcription regulation is still far from clear. Overall, I find that the statements made in the paper regarding the role of condensates to be on the strong site, and I recommend a bit more caution and consider alternative models, especially since the current manuscript does not show any experimental evidence on this topic.

In box 1, it would help the reader if parameter k and epsilon are explained.

In summary, the data is solid, but the interpretation may have significant flaws. To draw these conclusions, several additional experiments would have to be performed, preferably using more direct methods to measure to measure the kinetic parameters of bursting, and directly showing changes in the interaction between 3' and 5' of genes. In the present form, the conclusions drawn by the paper are not sufficiently backed up by data. The manuscript would require major revisions/analysis adjustments to be considered for publication.

**Reviewer 2**

**Are you able to assess all statistics in the manuscript, including the appropriateness of statistical tests used?**

No, I do not feel adequately qualified to assess the statistics.

**Comments to author:**

Transcription occurs in bursts- there is considerable interest in the understanding processes underlying bursting- as it provides a window into understanding the interplay between the many complex inputs into the transcriptional process. This study by the group of Hebenstreit explores the idea that bursting is determined, in part by coupling between the 5' and 3' ends of genes generating repeated transcript production in small time windows. The study, principally through modelling, then marries these ideas to the topical issue of LLPS.

The subject and hypothesis are very interesting, and the approach, to use flow to measure RNA FISH staining, is novel and more high throughput than other RNA measures, presumably allowing much better model fitting to RNA levels.

My major concern is that the modelling in the paper is based upon steady state transcript levels, not nascent RNA. The reduction in transcripts for the mutant alleles could easily be explained by altered kinetics of the decay process- which is to be expected anyway in a situation where the 3' end of the transcript carries a mutation. So the data may not be reporting transcription effects at all. I think this issue will be resolved by nascent RNA FISH.

Other points:

1. "More recently, it has been found that, in proximity to active genes, the PolIIs are incorporated in membrane-less droplets, maintained by a liquid-liquid phase separation (LLPS) from the rest of the nucleus, with the net effect of locally increasing the population of factors involved in initiation; when PolII is liberated from this domain, transcription can be initiated"

The data on LLPS and gene regulation functions are largely correlative. No functional data really exists, so I would caveat this section more. LLPS is potentially very interesting, and certainly topical, but its role has not been convincingly demonstrated as yet, so I would reduce the reference to it, certainly remove and explicit functional references.

"During a TetR blockade, PolIIs cannot transcribe and accumulate in the compartment." Does that imply that next to any actively repressed gene there is a fully packed compartment waiting to start transcribing? This view almost seems gives the gene some form of agency.

2. smFISH/FACS is a neat technique

3. artificial genes were used- this needs to be caveated heavily in the discussion.

4. Main issues:mutated HIV pA seems to have just as many problems with splicing than transcript count. To what extent are the effects on transcript count dependent on increased RNA turnover, rather than decreased transcription? This applies to all of Figure 1 (upon which the modelling is based). Ideally needs nascent RNAseq analysis. Even then- does an effect imply coupling, or an indirect effect on complex formation by titration of essential components- or physical blockage of new transcription?

5. "To gauge this background we deleted the env gene from its host cell line with Cas9 and performed the staining procedure as before."

This confused me- the env gene is viral is it not? How does the host cell have an env gene? Perhaps this should be explained more clearly.

6. Page 6, line 44 - significant difference between interaction scores of high-noise and intermediate-noise groups; Fig.4E - significant difference between interaction scores of high-noise and low-noise groups. What is the difference between text and the figure and why are panels 4D-E not referred to when mentioning the initial results in the text? Is the filtering out of zero-count genes making the difference (page 6, line 58)?

7. Page 8, line 52: "…while an alternative symmetric interpretation is possible, viz., that recycling permits higher expression at a given noise level"

Not sure this can be derived from the results, since the noise is lower at the higher levels of expression under same conditions.

8. Clerical/clarity issues:

a)	Order of supplementary figures and supplementary chapters doesn't always follow the text (e.g. Fig.S3, S1, S2)

b)	Fig.1A,C - Why is the TSS (position 0) marked after the start of the first exon?

c)	Fig.1A,C would be more intuitive if it was plotted on the same scale. The same for Fig.1B,D

d)	Fig.1E - Is there a drop in mRNA expression levels at high concentrations of Tet?

e)	Page 5, line 37 - "…. randomly sampled across the genome". Maybe I got this wrong: should this be randomly sampled only from the coding regions?

f)	Fig3.A and Fig.4C - legend box obscuring data points

g)	Fig.5A schematic - shouldn't the arrows describing the initiation (beta x (1-l)) and recycling (beta x l) point to TSS and TES, respectively?

**Reviewer 3**

**Are you able to assess all statistics in the manuscript, including the appropriateness of statistical tests used?**

No, I do not feel adequately qualified to assess the statistics.

**Comments to author:**

Cavallaro et al entitled "3'-5' crosstalk contributes to transcriptional bursting" investigates the contribution of Pol II recycling to bursting. The authors take advantage of two cell lines where integrated transgenes under the control of a Tet system have been characterised. Importantly, versions of these transgenes, containing a mutation in their respective pA signal have been characterized to show re-initiation defects. Here, the authors used these cell lines to measure transgene expression in single cells using a flow cytometry-based single molecule RNA FISH assay (flow-FISH) from which they extract bursting parameters using Bayesian modelling approaches. The data shows that pA mutants have more frequent but shorter bursts. Assuming that this effect of the pA mutation stems from its previously characterised recycling defect (see below), this suggests that 3'-5' Pol II recycling contributes to reducing transcription noise. The authors also analysed published sc-RNAseq and Pol II ChIA-PET datasets to suggest that a link between 3' 5' interactions (gene loops) and low noise can be observed genome-wide. Finally, they build a model of transcription that includes the concept of pol II recycling and molecular condensates.

The manuscript is well written and addresses an interesting question. It is also timely as transcriptional bursting is emerging as a key molecular mechanism for transcription regulation. Overall, I found the flow-FISH part more convincing than the genome-wide part, especially when combined with the modelling presented at the end. My detailed comments:

-	An overall comment is that the effect of the pA mutation on bursting is interpreted as a consequence of its effect on Pol II recycling (also called "3'-5' crosstalk" by the authors). While it is intuitive to interpret it that way, one can not exclude that the effects on bursting are a consequence of the termination defect. The authors should be more careful in differentiating the observations from their interpretations. The modelling presented at the end of the manuscript, provides evidence that recycling is indeed the mechanism, but until this section of the manuscript, the description should be that "pA mutation affects bursting, suggesting that recycling affect bursting but not ruling out that other effect of the mutation -such as termination defects- may contribute". The model at the end could in fact be introduced as a way to discriminate between these possible mechanisms. In those lines, it would be interesting to test alternative models as well, including one that includes termination but not recycling.

-	The model assumes that the recycled Pol II transits via the condensate before being re-loaded on the promoter during recycling. One can imagine that Pol II recycling is pathway for loading Pol II on promoters that bypasses the condensate. Can you test such a model and compare it with the current one. It would be interesting to see which one fits the data best.

-	The genome-wide analysis was less convincing to me. Indeed, the correlations between the "3'5' interaction score" and "v" or "Kon" (Figure 4DE) are very weak. In fact, one could not guess them from looking at the plots. They may be statistically significant due to the large number of measurements, but this does not mean they are biologically relevant. The poor correlation may stem from the fact that these values come from modelling of noisy data. Alternatively, there may not be such correlation between formation of a gene loop and noise. Indeed, one may imagine gene loops forming as default being in place to help recycling. There is no need for such a correlation for the existence of a functional role of recycling on bursting.

-	As a more minor point, Looking at Figure 1B,D, it looks to me as if HIS-env has a splicing defect in the pA mutant (that is: excess of intro reads relative to exons in the mutant, when compared to WT). Have you quantified this?

**Authors Response**

**Point-by-point responses to the reviewers’ comments:**

We would like to thank the reviewers for their efforts to study our manuscript and make constructive comments. We have now significantly revised and improved our manuscript based on the reviewers’ input.

Please find below a point-by-point response to the reviewers’ comments.

Reviewer #1

The manuscript of Cavallaro et al. studies the role of 3' - 5' crosstalk, gene looping and reinitiation of pol II in transcriptional bursting. This is an interesting concept, as the molecular mechanisms of bursting are mostly unknown. The hypothesis that the authors try to address experimentally has been suggested before, but has so far not been experimentally shown. The authors use a combination of approaches, including transgene analysis upon perturbation, genome-wide analysis of existing datasets and modeling, to show that reinitiation of Pol II upon termination contributes to bursting. The data quality is high, with many replicates and carefully performed controls. However, I have strong concerns on the analysis, interpretation and conclusions drawn from this data.

The authors use several transgene reporters and cell lines to study the effect of polyA mutations. Similar cell lines have been used before to show that polyA mutation reduce transcription initiations, which the authors also reproduce. The evidence that transcription initiation is really affected is not performed by the present study, but mostly relies on a previous study (ref 32). The authors then perform flowFISH to determine the mean and noise of the construct, which they then fit to several (bursting) models to acquire the bursting parameters. In general, it has been inherently difficult to capture dynamic parameters from indirect static measurements such as smFISH data, which is also true for flow-FISH

C1.1 My main concern is that polyA mutation, in addition to inhibiting transcription initiation, can significantly affect the stability of the transcript. If the readthrough transcripts are degraded much faster, or are not exported to the cytoplasm, this may significantly affect the distributions. I could not find any hints in the manuscript that the authors have addressed this option, or how it would affect their modelling results.

*R1.1 We did consider transcript stability. The half-lives of RNA produced from the HBB wt/mut gene, including the readthrough transcripts’, had been measured in Mapendano et al., Mol Cell 2010, and we adopted the reported figures for our paper. Further, we experimentally measured the env RNA half-life in our own lab. These data and considerations were reported in the supplementary information and used to adjust the bursting parameters plotted in Fig. 2.*

*To aid noticeability of this issue, we added the following text to the main manuscript (p5, ln286):*

*“Estimates of the degradation rates ̃ for both mutant and WT transgenes are listed in SI Appendix, section S5“*

*Incidentally, in the edited manuscript, we also included additional analyses to discuss the possible role of degradation rate in the expression noise, please see R1.7.*

*Following the reviewer comment we also checked changes in nuclear export and found that this was largely unaffected. The nuclear/cytoplasmic mRNA ratios were 0.370 and 0.366 for HBB cells at 250 ng mL−1 (medians over 43 and 40 cells, respectively), and 0.568 and 0.463 for HIV cells at 250 ng mL−1 (medians over 100 and 103 cells, respectively).*

*We added the following sentence to the main text (p4, ln237):*

*“Nuclear RNA export was largely unaltered by the mutations (Wilcoxon rank sum test on nuclear/cytoplasmic ratios from 83 HBB cells at 250 ng mL−1 Tet, P = 0.85; for 203 env cells P=6x10-9, but the ratios differed only by 10%). Note that FlowFISH data and their analysis/interpretation are unaffected by nuclear export issues.”*

C1.2	I therefore doubt that the effect on transcriptional noise that is observed can fully be explained by changes in bursting parameters. The authors would need to carefully exclude the option that there are effects on degradation or export of mRNA from the nucleus in these mutants.

*R1.2 Please see the point above.*

C1.3 extrinsic noise can also affect the distribution, one of which is cell cycle. The authors indeed nicely show that the contribution of cell cycle on the noise is very small. It is however a bit surprising that their overall extrinsic noise is such a small fraction of the total transcriptional noise, as it has been found to be much higher in other systems. I wonder whether other extrinsic noise components, such as cell size, could be missed in the current setup.

*R1.3 The reviewer correctly notices that in other systems the extrinsic noise was found to be higher, as we pointed out in the manuscript. We believe this to be due to the power of our FlowFISH system that allows studying intrinsic and extrinsic factors simultaneously, in great numbers of cells, and at great precision (see also R1.4).*

*Upon double-checking our numerical calculations, we found that a parameter (namely the average mRNA counts used to calibrate the flow cytometer reads at each cell-cycle stage) was slightly misspecified. Correction of this parameter slightly reduced extrinsic noise compared to what we previously reported. We implemented this correction in the new manuscript.*

*To further address the reviewer’s comment, we included the cell size as an additional extrinsic variable and repeated the calculations, which show that the extrinsic noise due to cell cycle and cell size remains a minor contributor to the total noise for HBB and env transgenes.*

*This analysis was added to Fig. 3 of the new manuscript.*

C1.4 Given these possible confounding effects of estimating transcription kinetics from such indirect measurements, I would find the results much more convincing if they were shown using more direct approaches. There are live-cell imaging assays available that could directly measure burst size and burst frequency in living cells, using MS2/PP7 transcription imaging, which would be compatible with the transgene approach.

*R1.4 It was our deliberate decision to use a flowFISH approach since this allowed us to adapt the transgene cell lines without any modifications and rely on the extremely thorough characterisation of these regarding 3’-5’ crosstalk in a previous study (Mapendano, Lykke-Andersen, Kjems, Bertrand, & Jensen, 2010). The second main reason we chose flowFISH is the large number of individual cells that can be recorded in a bias-free manner and which enabled us to examine population structures and evaluate extrinsic noise (see R1.3).*

*MS2/PP7 is a notoriously noisy and capricious assay that requires genetic introduction of 24 MS2 hairpin loops into the 3’ UTR of the gene of interest (Tutucci et al., 2018). This would extend the transgene lengths significantly and would potentially interfere with the 3’-5’ crosstalk. The cell lines edited in this way would need to be characterised and verified in all respects, essentially repeating the whole Mapendano et al., Mol. Cell 2010 study.*

*Please note that the other reviewers laud our flowFISH approach and its power and precision (and the latter is also echoed by this reviewer).*

C1.5 In addition to my concerns about the flowFISH interpretation, both the present manuscript, and the cited article 32, do not show that loss of the polyA site reduces contact between the 5' and 3' of a gene (minor note: ref 32 is wrongly cited in the introduction as if it shows this). Although there may be crosstalk, it appears that the conclusion that gene loops are involved in this is still not sufficiently shown experimentally.

*R1.5 We have rephrased the sentence that cites (Mapendano et al., 2010) (ref. [32] in the unrevised manuscript) to better reflect their actual result (p1, ln81):*

*“Importantly, it has been demonstrated that 3’-end processing favours transcription initiation; the presence of such 3’-5’ crosstalk in a gene increases its mean expression level [33].”*

C1.6 The authors continue to show that genes with more noise in scRNAseq data have more ChIA-PET interactions between 3' and 5' than genes with less noise. Although the effects are modest, but this is a conceptually interesting finding. I do wonder how specific this effect is for 5' - 3' interactions (loops), or if you would find similar higher interactions between the 5' and gene body. Given the recent insights from microC data (Hsieh et al, Mol Cell 2020), could it be possible that the promoter or (3' end) interacts along the entire gene, including the 3' end (as seen by the stripes in microC). From the current analysis, where the interaction of 5' and 3' are compared to random genomic locations with the same distance, this would not be clear. Would the authors see the same if the compared the interactions of promoter to gene body for high/low noise genes? If so, that would change the interpretation of their results.

*R1.6 Unsurprisingly, 5’ to 3’ interaction scores correlate with those for 5’ to gene body interactions; in fact, the higher the PolII abundance at a genomic position, the stronger its PolII-mediated interactions, and two proximal locations in a gene have similar amounts of PolII. This, in turn, implies that noise correlates with 5’ to gene body interaction scores, too. This observation does not change our results and we regard it as tangential to the paper’s focus. To acknowledge this, we added the following text (p6, ln387):*

*“Note that 5’ to 3’ interaction scores correlate with those for 5’ to gene body interactions; this appears unsurprising, given that spatial proximity at one location will favour interaction signals at neighbouring regions, and is tangential to our analyses.”*

C1.7 The modeling approach is a nice addition and shows that recycling of polymerases could contribute to bursting. Given my concerns with the interpretation of the experimental data, on its own this model does not prove that recycling occurs. Also, no other models without recycling are considered, and I wonder if there would be other models that would equally well explain the observed data.

*R1.7 We simulated and discussed two additional model settings in the new subsection “Alternative model settings” in line with the reviewer’s advice. Firstly, we considered a model with no recycling, where the degradation rate is not kept constant but can be increased to mimic this evident effect of the termination defects in the mutant gene. The results for this alternative model are not consistent with the observations (while they are consistent with single-cell expression data from influenza-infected cells – influenza is known to cause termination defects (Bauer et al., 2018; Russell, Trapnell, & Bloom, 2018; Zhao et al., 2018), please see the same subsection in our revised manuscript). See also R2.1 and R3.1.*

*Secondly, we considered a model which permits recycling, but doesn’t allow PolIIs to accumulate in a compartment before transcription begins. Again, the results deviate from the experimentally observed patterns, thus also ruling out this model as a good representation of the reality.*

*It is formally impossible to exclude the existence of a model with no recycling that reproduces the data from our transgenes. Nevertheless, our recycling model provides a parsimonious explanation for the observed pattern by tuning only one single parameter, which mirrors the minimal difference between the mutant and WT cell lines; therefore the recycling model remains a highly plausible representation of the biological reality.*

Minor comments:

C1.8 The hypothesis that liquid condensates are involved is interesting, but this is very much a developing field, where the role of phase separation in transcription regulation is still far from clear. Overall, I find that the statements made in the paper regarding the role of condensates to be on the strong side, and I recommend a bit more caution and consider alternative models, especially since the current manuscript does not show any experimental evidence on this topic.

*R1.8 We appreciate the reviewer’s word of caution. To soften our statements in this regard, we changed phrasing at several places:*

*p1, line 67: “[...] as the nuclear condensates are indeed able to restructure the genome, albeit results on LLPS are relatively preliminary at this stage.“*

*p1,	line 85: “The concept of LLPS is appears highly important in this regard [...]”*

*p2,	line 92: “We generically refer to the shuttling of PolIIs from 5’ to 3’, potentially passing through the LLPS compartment, as the recycling.”*

*P7, line 444: “[...] induction, polymerase flux between the LLPS droplet (or, more generically, a cluster of PolII [20]) and the gene, transcription, and decay, [...]”*

C1.9 In box 1, it would help the reader if parameter k and epsilon are explained.

*R1.9	We edited the last sentence of Box 1A) accordingly: “These models generate mRNA counts X and, in turn, fluorescence intensity, which also depends on the scaling factor and the measurement noise .*

Reviewer #2:

Transcription occurs in bursts- there is considerable interest in the understanding processes underlying bursting- as it provides a window into understanding the interplay between the many complex inputs into the transcriptional process. This study by the group of Hebenstreit explores the idea that bursting is determined, in part by coupling between the 5' and 3' ends of genes generating repeated transcript production in small time windows. The study, principally through modelling, then marries these ideas to the topical issue of LLPS.

The subject and hypothesis are very interesting, and the approach, to use flow to measure RNA FISH staining, is novel and more high throughput than other RNA measures, presumably allowing much better model fitting to RNA levels.

C2.1 My major concern is that the modelling in the paper is based upon steady state transcript levels, not nascent RNA. The reduction in transcripts for the mutant alleles could easily be explained by altered kinetics of the decay process- which is to be expected anyway in a situation where the 3' end of the transcript carries a mutation. So the data may not be reporting transcription effects at all. I think this issue will be resolved by nascent RNA FISH.

*R2.1 We have carefully considered the degradation of RNA in the original manuscript, please see R1.1. As a consequence, the dynamics we inferred actually relate to the kinetics of the transcription process itself (in contrast to downstream events).*

*Secondly, we agree with the referee that the reduction in transcripts for the mutant alleles could easily be explained by altered kinetics of the decay process, however, the observed noise patterns cannot. To demonstrate this, we performed more simulations (detailed in the new subsection “Alternative model settings” and studied single-cell expression data from influenza-infected cells, please see also R1.7 and C3.1)*

Other points:

C2.2 A.	"More recently, it has been found that, in proximity to active genes, the PolIIs are incorporated in membrane-less droplets, maintained by a liquid-liquid phase separation (LLPS) from the rest of the nucleus, with the net effect of locally increasing the population of factors involved in initiation; when PolII is liberated from this domain, transcription can be initiated"

The data on LLPS and gene regulation functions are largely correlative. No functional data really exists, so I would caveat this section more. LLPS is potentially very interesting, and certainly topical, but its role has not been convincingly demonstrated as yet, so I would reduce the reference to it, certainly remove and explicit functional references.

*R2.2 We have now significantly toned down LLPS-related statements. Please see R1.8.*

C2.3 B "During a TetR blockade, PolIIs cannot transcribe and accumulate in the compartment." Does that imply that next to any actively repressed gene there is a fully packed compartment waiting to start transcribing? This view almost seems gives the gene some form of agency.

*R2.3 Pools of promoter-associated PolII that are poised to start transcribing are very well established and are observed directly or indirectly in many different types of assays, such as imaging (Papantonis & Cook, 2013) or promoter peaks in ChIP-seq data.*

C2.4 2. smFISH/FACS is a neat technique

*R2.4 We thank the referee for this comment.*

C2.5 3. artificial genes were used- this needs to be caveated heavily in the discussion.

*R2.5 We edited the manuscript to state more clearly the use of transgenes in the first part of our study. In addition, we have included the following sentence to the discussion to acknowledge the issue:*

*P10, ln578: “[...] quantitative information available on the experimental device. Also, note that our transgenes might not exactly represent the average endogenous gene. Nevertheless, our setting is sufficient to resolve specific patterns [...]“*

*Note that our findings are not exclusively based on transgenes, but rather include genome-wide ChIA-PET data analyses and in depth explorations of in silico models.*

C2.6. Main issues:mutated HIV pA seems to have just as many problems with splicing than transcript count. To what extent are the effects on transcript count dependent on increased RNA turnover, rather than decreased transcription? This applies to all of Figure 1 (upon which the modelling is based). Ideally needs nascent RNAseq analysis. Even then- does an effect imply coupling, or an indirect effect on complex formation by titration of essential components- or physical blockage of new transcription?

*R2.6 The degradation rate of the mutated HIV transcript is enhanced (see R1.1), which does not affect our results (see R2.1), but means a relative increase of intronic sequencing reads (as part of the pre-mRNA) compared to exonic ones.*

*Nascent RNA has been analyzed in (Mapendano et al., 2010) and the authors also report that:*

*“Ratios for both intron 1- and intron 2-containing 1-globin pre-mRNA are equal between the 1 pA− and 1 pA+ cell lines within the first 2 hr of transcription induction (Figure S2B). Although splicing of the 1 pA− pre-mRNA is slightly affected 24 hr after induction, this phenotype occurs subsequent to depletion of RNAPII inside the gene and of TBP at the promoter. Thus, decreased splicing cannot be the primary cause of the mutant pA site-induced transcription defect."*

*Furthermore, we carried out a splicing analysis based on junction containing sequencing reads with the SGSeq software tool. No differences in splice events between wt and mut env RNA were found and spurious variants are supported by very few reads (~1). We have included these results in supplementary section S10E.*

C2.7 5. "To gauge this background we deleted the env gene from its host cell line with Cas9 and performed the staining procedure as before." This confused me- the env gene is viral is it not? How does the host cell have an env gene? Perhaps this should be explained more clearly.

*R2.7 env originally derives from the HIV genome and becomes integrated into DNA upon infection with the virus, but this is not the system we used. In fact, the env in our system is a transgene and was integrated into the HEK293 cell-line genome with the Flp-In system (Invitrogen). We then deleted the transgene from the same cell line to obtain an optimal control cell line without having to resort to another cell line that is similar in name only.*

C2.8 6. Page 6, line 44 - significant difference between interaction scores of high-noise and intermediate-noise groups; Fig.4E - significant difference between interaction scores of high-noise and low-noise groups. What is the difference between text and the figure and why are panels 4D-E not referred to when mentioning the initial results in the text? Is the filtering out of zero-count genes making the difference (page 6, line 58)?

*R2.8 The P-values in the text refer to the unfiltered-gene regression. P-values of the filtered regression lines were a bit higher (but still small) due to smaller sample size. To clarify this, we have edited the following sentence in the text (p7, ln429):*

*“Filtering out zero-count genes, for which there is little statistical information, increases the P-values to 2.0e-7, 2.0e-3, and 1.68e-5, respectively, due to smaller sample sizes, and yields the scatter plots of Fig. 4D-E“*

C2.9 7. Page 8, line 52: "...while an alternative symmetric interpretation is possible, viz., that recycling permits higher expression at a given noise level"

Not sure this can be derived from the results, since the noise is lower at the higher levels of expression under same conditions.

*R2.9 Please consider Fig.2A and Fig. 5B and imagine a horizontal line around CV2 equal to 1. Along this line, noise remains the same, while the high-recycling, wildtype cells admit to higher mean expression compared to the non-recycling (mut) cells. This is symmetric to fixing the mean expression, i.e., imagining a vertical line and noticing that different noise levels are allowed with the same average expression.*

C2.10 8. Clerical/clarity issues:

a)	Order of supplementary figures and supplementary chapters doesn't always follow the text (e.g. Fig.S3, S1, S2)

*R2.10 We have edited the references to the sections in the SI appendix accordingly.*

C2.11 b)	Fig.1A,C - Why is the TSS (position 0) marked after the start of the first exon? R2.11 We have corrected the TSS position on the figure based on the coverage data.

C2.12 c)	Fig.1A,C would be more intuitive if it was plotted on the same scale. The same for Fig.1B,D

*R2.12 As the mutant case has fewer reads than the WT, using the same scale would make the coverage plot for the mut genes smaller and thus less clear. The y-axes in the plots are clearly annotated in order to avoid confusion.*

C2.13 d)	Fig.1E - Is there a drop in mRNA expression levels at high concentrations of Tet?

*R2.13 This can be ascribed to experimental variation considering it varies among replicates (Please see fig. S2 in the SI appendix) and is taken into account by the Bayesian analysis.*

C2.14 e)	Page 5, line 37 - ".... randomly sampled across the genome". Maybe I got this wrong: should this be randomly sampled only from the coding regions?

*R2.14 Randomly sampling across the genome is correct, since PolII-ChIA-PET depends on both PolII abundance and genomic distance. This latter contribution is due to the fact that spatial proximity at one location will favour interaction. We simply normalised by the genomic distance in order to remove its contribution whilst keeping the PolII-mediated interactions.*

C2.15 f)	Fig3.A and Fig.4C - legend box obscuring data points

*R2.15 We have corrected these legend boxes accordingly.*

C2.16 g)	Fig.5A schematic - shouldn't the arrows describing the initiation (beta x (1-l)) and recycling (beta x l) point to TSS and TES, respectively?

*R2.16 We have slightly repositioned the arrows according to the reviewer’s suggestion. [beta (1¬l) is the transcription- not initiation rate].*

Reviewer #3:

[...] The manuscript is well written and addresses an interesting question. It is also timely as transcriptional bursting is emerging as a key molecular mechanism for transcription regulation. Overall, I found the flow-FISH part more convincing than the genome-wide part, especially when combined with the modelling presented at the end. My detailed comments:

C3.1 An overall comment is that the effect of the pA mutation on bursting is interpreted as a consequence of its effect on Pol II recycling (also called "3'-5' crosstalk" by the authors). While it is intuitive to interpret it that way, one can not exclude that the effects on bursting are a consequence of the termination defect. The authors should be more careful in differentiating the observations from their interpretations. The modelling presented at the end of the manuscript, provides evidence that recycling is indeed the mechanism, but until this section of the manuscript, the description should be that "pA mutation affects bursting, suggesting that recycling affect bursting but not ruling out that other effect of the mutation -such as termination defects- may contribute". The model at the end could in fact be introduced as a way to discriminate between these possible mechanisms. In those lines, it would be interesting to test alternative models as well, including one that includes termination but not recycling.

*R3.1 The term “3'-5' crosstalk” was established in a previous paper that characterized the cell lines in great detail with regards to precisely this issue. We are clear about this in the manuscript and cite this paper frequently (Mapendano et al, Mol Cell 2010).*

*Following the reviewer’s advice, we simulated alternative models where recycling is forbidden and the mRNA degradation rate can be tuned to explore its effect on the bursting parameters. The results are not compatible with the experimental observations (conversely to the recycling model); in fact, the pA mutation in the transgenes lowers both average expression levels and their noise, while increasing the degradation rate lowers the average expression but increases the noise.*

*In addition to that, we also analysed influenza-infected cells, as this infection causes termination defects, which gives results consistent with the simulations. These aspects are discussed and illustrated in the additional subsection “Alternative microscopic models” and Figure 6 of our revised manuscript. Please see also R1.7 and R2.1.*

C3.2 The model assumes that the recycled Pol II transits via the condensate before being re¬loaded on the promoter during recycling. One can imagine that Pol II recycling is pathway for loading Pol II on promoters that bypasses the condensate. Can you test such a model and compare it with the current one. It would be interesting to see which one fits the data best.

*R3.2 Following the reviewer’s comment we performed this test and discussed its results in the additional subsection titled “Alternative model settings”; we simulated a model where recycled PolII are not allowed to accumulate in a condensate. In contrast to our original model, this one yields results incompatible with the experimental observations, which therefore support our interpretation. The results are summarised in the new Fig. 6A (see also R1.7).*

C3.3 The genome-wide analysis was less convincing to me. Indeed, the correlations between the "3'5' interaction score" and "v" or "Kon" (Figure 4DE) are very weak. In fact, one could not guess them from looking at the plots. They may be statistically significant due to the large number of measurements, but this does not mean they are biologically relevant. The poor correlation may stem from the fact that these values come from modelling of noisy data. Alternatively, there may not be such correlation between formation of a gene loop and noise. Indeed, one may imagine gene loops forming as default being in place to help recycling. There is no need for such a correlation for the existence of a functional role of recycling on bursting.

*R3.3 The associations do appear clear to us even upon visual inspection, in particular Fig. 4F. As the reviewer suggests, they are highly significant according to statistical testing, a major purpose of which is precisely to avoid problems with visual inspection.*

*ChIA-PET and Drop-seq data are very well known for being subject to substantial amounts of technical noise, owing to cross-linking, proximity ligation and a highly complex and long protocol in the case of the former, and the single-cell nature of the latter. Despite these issues we observed significant associations, which we deem worth reporting.*

*Accepting the reviewer’s last point in this comment also would simply bolster our paper’s conclusions; it seems unnecessary to speculate on the absence of correlation even with ‘default gene loops to help recycling’, when in fact we do see significant correlations.*

C3.4 - As a more minor point, Looking at Figure 1B,D, it looks to me as if HIS-env has a splicing defect in the pA mutant (that is: excess of intro reads relative to exons in the mutant, when compared to WT). Have you quantified this?

*R3.3 Please see R2.6.*

*References*

*Bauer, D. L. V., Tellier, M., Martínez-Alonso, M., Nojima, T., Proudfoot, N. J., Murphy, S., & Fodor, E. (2018). Influenza Virus Mounts a Two-Pronged Attack on Host RNA Polymerase II Transcription. Cell Reports. https://doi.org/10.1016/j.celrep.2018.04.047*

*Mapendano, C. K., Lykke-Andersen, S., Kjems, J., Bertrand, E., & Jensen, T. H. (2010). Crosstalk between mRNA 3′ End Processing and Transcription Initiation. Molecular Cell, 40(3), 410–422. https://doi.org/10.1016/j.molcel.2010.10.012*

*Papantonis, A., & Cook, P. R. (2013). Transcription factories: Genome organization and gene regulation. Chemical Reviews, 113(11), 8683–8705. https://doi.org/10.1021/cr300513p*

*Russell, A. B., Trapnell, C., & Bloom, J. D. (2018). Extreme heterogeneity of influenza virus infection in single cells. ELife. https://doi.org/10.7554/eLife.32303*

*Tutucci, E., Vera, M., Biswas, J., Garcia, J., Parker, R., & Singer, R. H. (2018). An improved MS2 system for accurate reporting of the mRNA life cycle. Nature Methods. https://doi.org/10.1038/nmeth.4502*

*Zhao, N., Sebastiano, V., Moshkina, N., Mena, N., Hultquist, J., Jimenez-Morales, D., ... Marazzi, I. (2018). Influenza virus infection causes global RNAPII termination defects. Nature Structural and Molecular Biology. https://doi.org/10.1038/s41594-018-0124-7*

**Second round of review**

**Reviewer 3**

The authors have appropriately addressed my comments and concerns.
